# Acute Hepatitis E Virus Infection Triggering Autoimmune Hepatitis in a Patient With Chronic Liver Disease: Case Report and the Review of the Literature

**DOI:** 10.7759/cureus.56344

**Published:** 2024-03-17

**Authors:** Sayan Malakar, Nishant Shah, Ankit Mishra, Vipin Pandey, Vivek V Shirol, Naganath K Wodeyar, Prabhat Verma, Sai Prathap, Kartik Balankhe, Ramnawal Rao, Uday C Ghoshal

**Affiliations:** 1 Gastroenterology, Sanjay Gandhi Postgraduate Institute of Medical Sciences, Lucknow, IND; 2 Gastroenterology and Hepatology, Sanjay Gandhi Postgraduate Institute of Medical Sciences, Lucknow, IND; 3 Pathology, Sanjay Gandhi Postgraduate Institute of Medical Sciences, Lucknow, IND

**Keywords:** infectious hepatitis, transjugular liver biopsy, live cirrhosis, acute hepatitis e, autoimmune hepatitis with cirrhosis

## Abstract

Acute viral hepatitis E (HEV) is the most common form of acute viral hepatitis in India. It is associated with self-limiting disease in most cases. However, the chronic form of HEV is also being increasingly recognized. Other viral infections like the hepatitis A virus (HAV) have been implicated in inciting autoimmune hepatitis. HEV infection has been associated with the formation of circulating liver-directed autoantibodies, however autoimmune liver disease following acute HEV infection has been rarely reported. Here we present a case of a 72-year-old diabetic lady who presented to us with an asymptomatic rise of liver enzymes. Investigations suggested metabolic dysfunction associated with steatotic liver disease. After three months of the diagnosis, she developed acute-on-chronic liver failure and her anti-HEV came out positive. She was managed accordingly. Afterwards patient had persistent high liver enzymes, so she underwent a liver biopsy. Her liver biopsy was compatible with autoimmune hepatitis.

## Introduction

Autoimmune hepatitis (AIH) is an uncommon form of chronic liver disease that mainly affects female patients [1]. Breakdown of self-tolerance, molecular mimicry, and autoimmunity are the proposed mechanisms of autoimmune hepatitis, however [2]. Viral hepatitis like acute viral hepatitis A (HAV) has been implicated in the pathogenesis of AIH [3]. Emerging data suggest that acute hepatitis E (HEV) infection may trigger AIH [4].

Here we present a case report of HEV-induced AIH in a case of chronic liver disease (CLD). A 72-year-old lady with diabetes and hypertension presented with an asymptomatic rise of liver enzymes. She was diagnosed with metabolic dysfunction associated-steatotic liver disease (MASLD). Then the patient developed acute-on-chronic liver failure (ACLF) three months after the initial diagnosis. The patient’s IgM-anti-HEV was found to be positive. All other alternative etiologies were ruled out. The patient improved after conservative management. On follow-up, her liver enzymes were persistently high. She developed circulating liver-directed autoantibodies like anti-nuclear antibodies (ANA) and anti-smooth muscle autoantibodies (ASMA). She underwent a liver biopsy which was suggestive of AIH. The patient was managed with steroids, and she went into remission after three months of therapy. This is one of the few case reports of acute HEV infection triggering AIH in a patient with chronic liver disease.

## Case presentation

**Figure 1:**
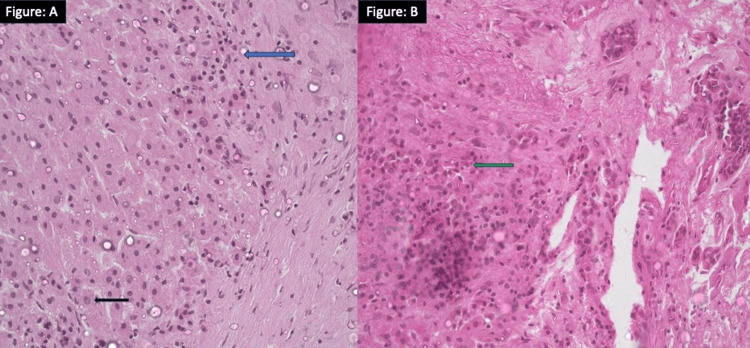
A: Hepatocytes show mild lymphoplasmacytic interface hepatitis (blue arrow) and occasional foci of emperipolesis (black arrow). (H&E stain 40x magnification) B: Portal tracts are expanded by marked fibrosis, and lymphoplasmacytic inflammation (green arrow). (H&E stain 20x magnification) H&E: hematoxylin and eosin

The 72-female diabetic and hypertensive patient presented with incidentally detected elevated serum liver enzymes. Her ultrasound abdomen revealed coarse echotexture, an irregular outline of the liver. Her portal vein diameter was 16 mm (normal < 13 mm). As she had metabolic risk factors (diabetes, hypertension, body mass index of 25.6 kilograms/meter square) possibility of MASLD (previously known as non-alcoholic fatty liver disease) was kept. All other alternate etiologies were ruled out (Table 1). She had no evidence of any other liver-related decompensation. A liver biopsy was planned three months after the initial diagnosis she presented to us with jaundice with prodromal features, ascites, and altered sensorium. Her total bilirubin was 17.2 mg/dL (conjugated 12.2 mg/dL) with high aspartate aminotransferase (AST) and alanine aminotransferase (ALT) levels. She was diagnosed with ACLF. For the etiological evaluation of the acute precipitant of her liver injury, necessary investigations were done. Immunoglobulin (Ig) M anti-HEV came out to be positive and her HEV ribonucleic acid (RNA) by real-time polymerase chain reaction (PCR) was 18200 copies/milliliter. Other viral markers including anti-hepatitis C antibody, HCV RNA, hepatitis B viral deoxyribonucleic acid (HBV DNA), IgM anti-HBc, and IgM anti-HAV were negative. The patient was managed conservatively. She was discharged after 19 days of hospitalization. On follow-up, her ascites and hepatic encephalopathy resolved but bilirubin and liver enzyme levels were persistently high. After seven months of her discharge, her bilirubin was 5.2 mg/ dL with high AST and ALT (AST 112 Unit/L and ALT 101 U/L). This time patient was found to have liver-directed autoantibodies with a high IgG level of 2020 mg/dL (normal <1800 mg/dL). Her ANA and ASMA were positive. Because of persistent hepatitis, she was posted for a transjugular liver biopsy. Her trans jugular liver biopsy revealed lymphoplasmacytic infiltration, emperipolesis, and moderate interface hepatitis suggestive of AIH (Figure 1A, 1B).

**Table 1:** Laboratory features of the patients before, during, and after the episode of acute viral hepatitis E AST: aspartate aminotransferase; ALT: alanine aminotransferase; ALP: alkaline phosphatase; Ig: immunoglobin; ANA: anti-nuclear antibody; ASMA: anti-smooth muscle antibody; AMA: anti-mitochondrial antibody; HEV; hepatitis E virus; RT-PCR: reverse-transcriptase polymerase chain reaction

Patient characteristics	Before acute viral hepatitis E	During acute viral hepatitis E	On follow-up after seven months	Twelve months after acute viral hepatitis E
Hemoglobin (g/dL)	10.1	9.2	9.0	9.8
Total leukocyte count (per/mm^3^)	4900	5400	6500	7000
Platelet count (lacs/mm^3^)	1.66	1.50	1.34	2.01
Total Bilirubin mg/dL	1.6	17.2	5.2	1.44
Conjugated Bilirubin	1.0	12.2	3.2	1.01
AST (U/L)	82	303	112	43
ALT (U/L)	97	337	110	58
ALP (U/L)	178	430	259	118
Albumin (g/dL)	3.4	2.9	3.1	4.4
Protein	7.2	5.78	7.2	7.8
IgG (<1800 mg/dL)	1060	Not done	2020	1620
ANA	Negative	Not done	4+ (1:100)	4+ (1:100)
ASMA	Negative	Not done	2+ (1:40)	2+ (1:40)
Anti-LKM-1	Negative	Not done	Negative	Negative
AMA	Negative	Not done	Negative	Negative
IgM Anti HEV	Not done	Positive	Negative	Negative
HEV RNA RT-PCR	Not done	18200 copies/mL	Not detected	Not detected

Reverse-transcriptase polymerase chain reaction (RT-PCR) for HEV RNA was also done as we planned to start the patient on oral prednisolone. Her HEV RNA was not detected this time.

**Figure 2:**
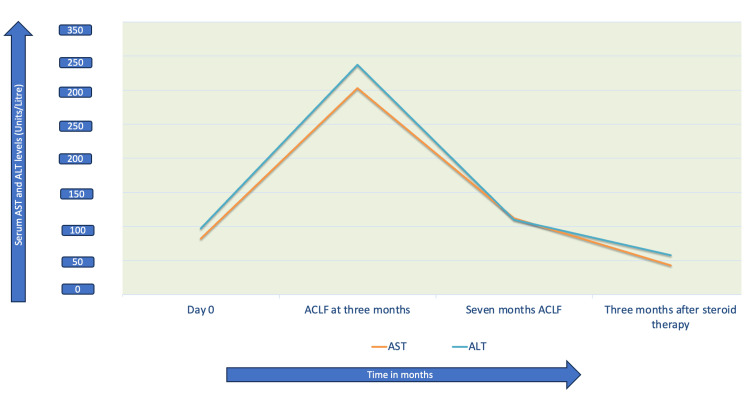
Trends of liver function tests over time ACLF: acute on chronic liver failure; ALT: alanine aminotransferase; AST: aspartate aminotransferase

The patient was started on 40 mg oral prednisolone. After six weeks of treatment, her liver enzymes came down and the steroid was tapered to five mg per day over the next four months (Figure 2).

## Discussion

AIH is an uncommon cause of chronic liver disease and viral infections are known to trigger AIH [5]. Different viral and bacterial infections are known to trigger autoimmunity [6,7]. They are associated with different various rheumatological diseases [7,8]. HEV is classically associated with circulating liver-directed autoantibodies. Reports by Gui et al. found that following acute HEV, 7.7% of patients may have circulating anti-mitochondrial antibody (AMA) or ANA [9]. Another study by Piccoli et al. [9] found that acute HEV infection may present with circulating autoantibodies. Among 48 patients with acute viral hepatitis half of them had circulating liver-directed autoantibodies. The most common autoantibody was ANA (33%) followed by ASMA (21%) and antineutrophil cytoplasmic antibody (ANCA) (14.6%). However, there was no evidence of autoimmune liver injury associated with these antibodies. After 12 months of follow-up majority of them came out to be negative for the antibodies. A study from China [10] revealed that about 37% of patients may have persistent circulating following acute HEV but none of them developed AIH. Conversely, there are higher prevalence of HEV infections among patients with AIH. Around 7% of patients with AIH have circulating antibodies against HEV. Cryoglobulinemia is also associated with HEV infections. It is present in around 7-27% of patients with HEV infections but without any clinical manifestations except neurological involvement [11]. Autoimmune liver diseases are often associated with circulating antibodies including immunoglobulin G-4, which can be found in acute viral hepatitis as well [12,13].

Most of the works of literature have documented the circulating autoantibodies following HEV infections. Only a few case reports are available where acute HEV infection manifests as liver injury mimicking AIH (Table 2). 

**Table 2:** Cases of acute viral hepatitis E with autoimmune hepatitis IAIHG: Autoimmune Hepatitis Group; AIH: autoimmune hepatitis; AMA: anti-mitochondrial antibody; ANA: anti-nuclear antibody; ASMA: anti-smooth muscle antibody; HEV; hepatitis E virus; Ig: immunoglobin

References	No. of patients	Age/Gender	IgG	Autoantibody	HEV RNA/IgM	AIH score	Treatment
AIH following acute viral hepatitis. Elfert et al. [4]	1	36 years/Female	1690 g/L	ANA: 1:640	N/A	IAIHG score of 23	Treated with azathioprine and steroid. Responded to treatment
Acute HEV infection mimicking flare of AIH. Calisti et al. [14]	1	44 years/Female	IgG 1940 g/L	ASMA: Positive	3.9 log10 IU/mL	IAIHG score: 7	Managed with prednisolone and azathioprine
Acute HEV with concomitant AIH. Patel et al. [15]	1	32 years/Male	N/A	ANA: 1:1280 + ASMA: 160+ AMA: 1:40+	IgM HEV: Positive	IAIHG score: 13	Improved with conservative treatment

Case reports of acute hepatitis and autoimmune hepatitis

A total of three cases have been described with patients with acute viral hepatitis E mimicking AIH (Table 2).

Elfert et al. reported a case of a 36-year-old female with systemic lupus erythematosus (SLE) who presented with acute viral hepatitis E [4]. Like our case. as she was not improving on conservative treatment, she underwent further investigations. She had circulating autoantibodies and liver biopsies were also suggestive of AIH. The other two patients had concomitant acute viral hepatitis with features of AIH. All patients were successfully managed with immunosuppressives.

Our case presented similarly as she had baseline metabolic syndrome (diabetes, dyslipidemia, and obesity) and presented with MASLD. Her initial workup for AIH was negative. Following acute HEV-related ACLF, she developed AIH. She was successfully managed with steroid and immunosuppressive.

One of the strengths of our report is that we have ruled out chronic HEV before steroid therapy. It is imperative to diagnose AIH after ruling out acute HEV infection as AIH is often associated with long-term consequences including cirrhosis and hepatocellular carcinoma [16,17]. We have a few limitations as it is a case report. We did not perform a liver biopsy before the onset of HEV-ACLF as she fulfilled the diagnostic criteria of MASLD. This is one of the few case reports of AIH triggered by HEV infection. 

## Conclusions

Acute HEV and HAV infections are known to incite AIH. The presence of serum autoantibody should be interpreted cautiously in a patient with acute HEV infection. Acute HEV infection should be ruled out in patients with AIH in appropriate clinical settings.

## References

[1] Choudhuri G, Somani SK, Baba CS, Alexander G (2005). Autoimmune hepatitis in India: profile of an uncommon disease. BMC Gastroenterol.

[2] Sucher E, Sucher R, Gradistanac T, Brandacher G, Schneeberger S, Berg T (2019). Autoimmune hepatitis-immunologically triggered liver pathogenesis-diagnostic and therapeutic strategies. J Immunol Res.

[3] Singh G, Palaniappan S, Rotimi O, Hamlin PJ (2007). Autoimmune hepatitis triggered by hepatitis A. Gut.

[4] Elfert KA, Qasim HM, Faisal MM, Elghazali A, Siddiqui MY, Petkar M, Sadik N (2021). Hepatitis E viral association with autoimmune hepatitis: a viral trigger or cross-reactivity. Case Rep Gastroenterol.

[5] Terziroli Beretta-Piccoli B, Ripellino P, Gobbi C (2018). Autoimmune liver disease serology in acute hepatitis E virus infection. J Autoimmun.

[6] Di Bartolomeo S, Carubbi F, Cipriani P (2020). Hepatitis E virus and rheumatic diseases: what do rheumatologists need to know?. BMC Rheumatol.

[7] Malakar S, Sharma TD, Raina S, Sharma KN, Kapoor D (2019). Guillain Barre syndrome with pulmonary tuberculosis: a case series from a tertiary care hospital. J Family Med Prim Care.

[8] Fousekis FS, Mitselos IV, Christodoulou DK (2020). Extrahepatic manifestations of hepatitis E virus: an overview. Clin Mol Hepatol.

[9] Gui H, Wang W, Li Q, Li Z, Lu J, Xie Q (2021). Autoimmune liver disease-associated serologic profiling in Chinese patients with acute hepatitis E virus infection. Immunol Res.

[10] Wu J, Guo N, Zhu L (2020). Seroprevalence of AIH-related autoantibodies in patients with acute hepatitis E viral infection: a prospective case-control study in China. Emerg Microbes Infect.

[11] Horvatits T, Schulze Zur Wiesch J, Polywka S (2020). Significance of anti-nuclear antibodies and cryoglobulins in patients with acute and chronic HEV infection. Pathogens.

[12] Malakar S, Mishra P, Paturu R, Verma R, Ghoshal UC (2023). Primary sclerosing cholangitis with high immunoglobulin-G4. J Hepatol.

[13] Santos VC, Schinoni MI, Oliveira IS, Atta ML, Atta AM (2019). IgG1 and IgG4 antibodies against Core and NS3 antigens of hepatitis C virus. Rev Soc Bras Med Trop.

[14] Calisti G, Irish DN, Ijaz S, Tedder RS, Moore K (2017). Acute hepatitis E mimicking a flare of disease in a patient with chronic autoimmune hepatitis. Ann Hepatol.

[15] Patel I, Ching Companioni R, Bansal R, Vyas N, Catalano C, Aron J, Walfish A (2016). Acute hepatitis E presenting with clinical feature of autoimmune hepatitis. J Community Hosp Intern Med Perspect.

[16] Malakar S, Pande G, Mishra P, Ghoshal UC (2024). Incidence of hepatocellular carcinoma in patients with autoimmune liver disease in India. J Clin Exp Hepatol.

[17] Malakar S, Mohindra S, Mishra P (2024). Implications of gender on the outcome in patients with autoimmune hepatitis. Cureus.

